# Supplementary material for: Understanding the treatment burden of people with chronic conditions in Kenya: A cross-sectional analysis using the Patient Experience with Treatment and Self-Management (PETS) questionnaire
Source: PLOS Glob Public Health. 2023 Jan 17;3(1):e0001407. doi: 10.1371/journal.pgph.0001407 (PMC10021888; doi:10.1371/journal.pgph.0001407)
Supplement: S1 Table — (DOCX) [file pgph.0001407.s002.docx]

## **S1 Table. Frequency of PETS domain items, total sample and by county: Medical information, medication, medical appointments, monitoring health and medical and health care expenses**

|  | **% rating item as difficult or very difficult (N)** | | |
| --- | --- | --- | --- |
|  | **Total sample** | **Busia** | **Trans Nzoia** |
| **Medical information** *(n=301)* |  |  |  |
| Learn about health problem(s) | 23.9% (72) | 28.0% (42) | 19.9% (30) |
| Learn what foods to eat | 23.6% (71) | 27.3% (41) | 19.9% (30) |
| Find information on medications to take | 19.6% (59) | 18.7% (28) | 20.5% (31) |
| Understand changes to treatment plan | 15.3% (46) | 21.3% (32) | 9.3% (14) |
| Understand reasons for taking medication | 9.6% (29) | 11.3% (17) | 7.9% (12) |
| Find trusted sources of medical information | 18.6% (56) | 14.0% (21) | 23.2%(35) |
| Understand advice from different providers | 14.0% (42) | 18.0% (27) | 10.0% (15) |
| **Medications** *(n=295)* |  |  |  |
| Organise medicines | 5.3% (16) | 9.0% (13) | 2.0% (3) |
| Take more than one medicine every day | 7.3% (22) | 12.4% (18) | 2.7% (4) |
| Take medicines several times each day | 8.6% (26) | 13.8% (20) | 4.0% (6) |
| Refill medicines | 23.9% (72) | 23.4% (34) | 25.3% (38) |
| Adjust medicines | 11.3% (34) | 18.6% (27) | 4.7% (7) |
| Take medicines as directed | 4.0% (12) | 4.1% (6) | 4.0% (6) |
| Plan daily activities around medicine schedule | 8.6% (26) | 7.6% (11) | 10.6% (16) |
| **Medical appointments** *(n=301)* |  |  |  |
| Make or keep medical appointments | 14.3% (43) | 16.0% (24) | 12.6% (19) |
| Schedule and keep track of medical appointments | 12.6% (38) | 14.7% (22) | 10.6% (16) |
| Make or keep medical appointment with different providers | 15.9% (48) | 20.7% (31) | 11.3% (17) |
| Find time to get to medical appointment | 8.6% (26) | 10.7% (16) | 6.6% (10) |
| Find energy to get to medical appointment | 9.0% (27) | 10.0% (15) | 7.9% (12) |
| Find transport to get to medical appointment | 37.9% (114) | 48.0% (72) | 27.8% (42) |
| **Monitoring health** |  |  |  |
| Monitor health behaviours (n=301) | 23.60% (71) | 31.3% (47) | 15.9% (24) |
| Monitor health condition (n=296) | 44.5% (134) | 56.7% (85) | 32.5% (49) |
| **Medical and health care expenses** *(n=301)* |  |  |  |
| Plan for future because of medical expenses | 55.1% (166) | 61.3% (92) | 49.0% (74) |
| Pay for healthy foods | 53.2% (160) | 62.0% (93) | 44.4% (67) |
| Pay for all medical expenses | 58.5% (176) | 67.3% (101) | 49.7% (75) |
| Pay for medicines | 54.2% (163) | 64.7% (97) | 43.7% (66) |
| Understand what is covered by health insurance * | 41.9% (126) | 40.0% (60) | 43.7% (66) |

* n=69 (22.9%) responding ‘does not apply to me’ (Busia: 21%; Trans Nzoia: 25%)
