# Supplementary material for: Understanding the treatment burden of people with chronic conditions in Kenya: A cross-sectional analysis using the Patient Experience with Treatment and Self-Management (PETS) questionnaire
Source: PLOS Glob Public Health. 2023 Jan 17;3(1):e0001407. doi: 10.1371/journal.pgph.0001407 (PMC10021888; doi:10.1371/journal.pgph.0001407)
Supplement: S2 Table — (DOCX) [file pgph.0001407.s003.docx]

##

## **S2 Table. Mean score and frequency of PETS domain items: Diet, exercise and physical therapy, and difficulty with health services**

|  | **% agreeing or strongly agreeing with statement (N)** | | |  |
| --- | --- | --- | --- | --- |
|  | **Total sample** | **Busia** | **Trans Nzoia** | |
| **Diet** *(n=279)* |  |  |  | |
| Having to give up too many foods | 63.8% (192) | 75.9% (104) | 62.0% (88) | |
| Difficulty finding healthy foods | 54.5% (164) | 68.6% (94) | 49.3% (70) | |
| Hard to follow provider recommendations for healthy eating | 46.8% (141) | 51.8% (71) | 49.3% (70) | |
| **Exercise and physical therapy** |  |  |  | |
| Difficulty finding time to exercise/do physical therapy *(n=271)* | 25.2% (76) | 30.8% (40) | 25.5% (36) | |
| Difficulty following provider recommendations for exercise/physical therapy *(n=270)* | 30.2% (91) | 36.2% (47) | 31.4% (44) | |
| Difficulty getting motivated to do exercise/physical therapy *(n=270)* | 30.6% (92) | 36.2% (47) | 32.1% (45) | |
| Physical pain & discomfort limits ability to exercise/do physical therapy *(n=270)* | 49.8% (150) | 63.1% (82) | 48.8% (68) | |
| **Difficulty with health care services** *(n=301)* |  |  |  | |
| Different providers not communicating *^(a)^* | 14.0% (42) | 20.7% (31) | 7.3% (11) | |
| Seeing too many different specialists *^(b)^* | 22.6% (68) | 34.7% (52) | 10.6% (16) | |
| Filling out forms relating to health care *^(c)^* | 19.9% (60) | 18.7% (28) | 21.1% (32) | |
| Getting appointments at convenient times *^(d)^* | 13.6% (41) | 12.0% (18) | 15.2% (23) | |
| Getting specialist appointments *^(e)^* | 14.0% (42) | 14.0% (21) | 13.9% (21) | |
| Long wait at medical appointments *^(f)^* | 29.6% (89) | 36.7% (55) | 22.6% (34) | |
| Long wait at pharmacy for medicines | 22.3% (67) | 30.0% (45) | 14.5% (22) | |

^(a)^ % responding ‘does not apply to me’: 37.5% (n=113) (Busia: 59%; Trans Nzoia: 17%); ^(b)^ 34.9% (105) (Busia: 52%; Trans Nzoia: 18%); ^(c)^ 25.5% (n=76) (Busia: 31%; Trans Nzoia: 19%); ^(d)^ 15.3% (n=46) (Busia: 13%; Trans Nzoia: 17%); ^(e)^ 26.9% (n=81) (Busia: 37%; Trans Nzoia: 17%); ^(f)^ 6.6% (n=20) (Busia: 5; Trans Nzoia: 9%)
