# Supplementary material for: Understanding the treatment burden of people with chronic conditions in Kenya: A cross-sectional analysis using the Patient Experience with Treatment and Self-Management (PETS) questionnaire
Source: PLOS Glob Public Health. 2023 Jan 17;3(1):e0001407. doi: 10.1371/journal.pgph.0001407 (PMC10021888; doi:10.1371/journal.pgph.0001407)
Supplement: S3 Table — (DOCX) [file pgph.0001407.s004.docx]

##

## **S3 Table. Mean score and frequency of PETS domain items: Reliance on medicine, side effects of medicine, relationship with others**

|  | **% responding to be quite or very bothered by issue (N)** | | | ***% responding to be somewhat, quite or very bothered by issue (N)*** | | |
| --- | --- | --- | --- | --- | --- | --- |
|  | **Total sample** | **Busia** | **Trans Nzoia** | ***Total sample*** | ***Busia*** | ***Trans Nzoia*** |
| **Reliance on medicine** *(n=295)* | 12.6% (38) | 18.0% (27) | 7.7%  (11) | *30.6% (92)* | *31.7% (46)* | *30.7% (46)* |
| **Side effects of medicine** *(n=295)* | 12.0% (36) | 18.7% (28) | 5.3%  (8) | *31.2% (94)* | *47.2% (54)* | *26.5% (40)* |
| **Relationship with others** *(n=301)* |  |  |  |  |  |  |
| Feeling dependent on others for health care needs | 20.3% (61) | 30.7% (46) | 9.9%  (15) | *36.2% (109)* | *46.0% (69)* | *26.5% (40)* |
| Others reminding you to do things for health | 8.3%  (25) | 14.0% (21) | 2.6%  (4) | *19.3% (58)* | *26.0% (39)* | *12.6% (19)* |
| Health care needs creating tensions in relationships | 15.9% (48) | 26.7% (40) | 5.3%  (8) | *29.2% (88)* | *42.7% (64)* | *15.9% (24)* |
| Others not understanding health situation | 15.9% (48) | 24.0% (36) | 8.0%  (12) | *28.2% (85)* | *34.7% (52)* | *22.5% (34)* |
