# Supplementary material for: Understanding the treatment burden of people with chronic conditions in Kenya: A cross-sectional analysis using the Patient Experience with Treatment and Self-Management (PETS) questionnaire
Source: PLOS Glob Public Health. 2023 Jan 17;3(1):e0001407. doi: 10.1371/journal.pgph.0001407 (PMC10021888; doi:10.1371/journal.pgph.0001407)
Supplement: S4 Table — (DOCX) [file pgph.0001407.s005.docx]

##

## **S4 Table. Mean score and frequency of PETS domain items: Role/social activity limitations due to self-management**

|  | **% responding 'quite a bit' or 'very much' (N)** | | | ***% responding 'somewhat', quite a bit' or 'very much' (N)*** | | |
| --- | --- | --- | --- | --- | --- | --- |
|  | **Total sample** | **Busia** | **Trans Nzoia** | ***Total sample*** | ***Busia*** | ***Trans Nzoia*** |
| **Role/social activity limitations due to self-management** *(n=301)* |  |  |  |  |  |  |
| Interference with work | 29.9% (90) | 41.3% (62) | 18.5% (28) | *40.2% (121)* | *46.7% (70)* | *33.8% (51)* |
| Interference with family responsibilities | 26.6% (80) | 38.7% (58) | 14.7% (22) | *35.6% (107)* | *42.7% (64)* | *28.5% (43)* |
| Interference with daily activities | 26.9% (81) | 38.7% (58) | 15.2% (23) | *40.2% (121)* | *47.3% (71)* | *33.1% (50)* |
| Interference with hobbies/leisure activities | 17.9% (54) | 25.3% (38) | 10.6% (16) | *26.2% (79)* | *32.0% (48)* | *20.5% (31)* |
| Interference with ability to spend time with family | 13.3% (40) | 22.6% (34) | 4.0%  (6) | *20.9% (63)* | *28.7% (43)* | *13.2% (20)* |
| Interference travel for work/vacation | 16.6% (50) | 25.3% (38) | 7.9%  (12) | *26.6% (80)* | *30.7% (46)* | *22.5% (34)* |
