# Supplementary material for: Understanding the treatment burden of people with chronic conditions in Kenya: A cross-sectional analysis using the Patient Experience with Treatment and Self-Management (PETS) questionnaire
Source: PLOS Glob Public Health. 2023 Jan 17;3(1):e0001407. doi: 10.1371/journal.pgph.0001407 (PMC10021888; doi:10.1371/journal.pgph.0001407)
Supplement: S5 Table — (DOCX) [file pgph.0001407.s006.docx]

##

## **S5 Table. Mean score and frequency of PETS domain items: Physical/mental exhaustion**

|  | **% responding 'often' or 'always' (N)** | | | ***% responding 'sometimes', 'often' or 'always' (N)*** | | |
| --- | --- | --- | --- | --- | --- | --- |
|  | **Total sample** | **Busia** | **Trans Nzoia** | ***Total sample*** | ***Busia*** | ***Trans Nzoia*** |
| **Physical/mental exhaustion** *(n=301)* |  |  |  |  |  |  |
| Feeling angry | 12.0% (36) | 18.7% (28) | 5.3%  (8) | *61.8% (186)* | *58.0% (87)* | *65.6% (99)* |
| Feeling preoccupied | 12.3% (37) | 18.0% (27) | 6.6%  (10) | *59.5% (179)* | *60.0% (90)* | *58.3% (88)* |
| Feeling sad or depressed | 10.3% (31) | 16.6% (25) | 4.0%  (6) | *58.1% (175)* | *56.7% (85)* | *59.6% (90)* |
| Feeling worn out | 16.3% (49) | 24.6% (37) | 7.9%  (12) | *68.8% (207)* | *66.0% (99)* | *71.5% (108)* |
| Feeling frustrated | 7.6%  (23) | 11.3% (17) | 4.0%  (6) | *53.1% (160)* | *48.0% (72)* | *58.3% (88)* |
