# Supplementary material for: Understanding the treatment burden of people with chronic conditions in Kenya: A cross-sectional analysis using the Patient Experience with Treatment and Self-Management (PETS) questionnaire
Source: PLOS Glob Public Health. 2023 Jan 17;3(1):e0001407. doi: 10.1371/journal.pgph.0001407 (PMC10021888; doi:10.1371/journal.pgph.0001407)
Supplement: S6 Table — (DOCX) [file pgph.0001407.s007.docx]

##

## **S6 Table. Mean PETS domain scores by socio-demographic and health-related characteristics**

|  | **Medical information** | **Medications** | **Medical appoint-ments** | | **Monitoring health** | **Interpersonal challenges** | | | **Medical & health care expenses** | | | | **Difficulty with health care services** | | | | **Role/social activity limitations** | | | **Physical/ mental exhaustion** | | | **Bother/ medicine reliance** | | | | **Bother/ medicine side effects** | | | | **Diet** | | | | **Exercise/ physical therapy** |
| --- | --- | --- | --- | --- | --- | --- | --- | --- | --- | --- | --- | --- | --- | --- | --- | --- | --- | --- | --- | --- | --- | --- | --- | --- | --- | --- | --- | --- | --- | --- | --- | --- | --- | --- | --- |
|  | Mean (SD) | Mean (SD) | Mean (SD) | | Mean (SD) | Mean (SD) | | | Mean (SD) | | | | Mean (SD) | | | | Mean (SD) | | | Mean (SD) | | | Mean (SD) | | | | Mean (SD) | | | | Mean (SD) | | | | Mean (SD) |
| ***Age*** |  |  |  | |  |  | | |  | | | |  | | | |  | | |  | | |  | | | |  | | | |  | | | |  |
| <60 years | 33.9 (21.1) | 24.0 (16.8) | 33.7 (20.3) | | 48.8 (25.7) | 24.2 (25.2) | | | 63.0 (22.1) | | | | 37.1 (19.5) | | | | 31.6 (28.1) | | | 40.8 (21.3) | | | 28.6 (33.4) | | | | 30.0 (30.7) | | | | 61.7 (25.3) | | | | 41.1 (26.1) |
| 60+ years | 31.9 (21.1) | 23.8 (20.2) | 29.5 (19.8) | | 48.6 (27.9) | 23.1 (26.9) | | | 63.0 (24.3) | | | | 34.8 (18.6) | | | | 32.8 (26.9) | | | 38.4 (20.6) | | | 23.4 (29.0) | | | | 22.4 (29.4) | | | | 53.3 (25.5) | | | | 46.4 (25.3) |
| *p value* | *p=0.415* | *p=0.918* | *p=0.075* | | *p=0.925* | *p=0.717* | | | *p=0.988* | | | | *p=0.344* | | | | *p=0.689* | | | *p=0.331* | | | *p=0.169* | | | | ***p=0.033*** | | | | ***p=0.003*** | | | | *p=0.102* |
| ***Gender*** |  |  |  | |  |  | | |  | | | |  | | | |  | | |  | | |  | | | |  | | | |  | | | |  |
| Female | 34.5 (21.0) | 24.3 (17.4) | 32.3 (19.7) | | 49.9 (25.2) | 24.0 (25.7) | | | 65.3 (20.8) | | | | 36.7 (19.2) | | | | 29.9 (26.1) | | | 41.3 (20.8) | | | 26.9 (32.1) | | | | 27.1 (29.6) | | | | 59.2 (26.5) | | | | 44.1 (25.8) |
| Male | 30.5 (21.6) | 23.5 (20.3) | 31.0 (21.1) | | 47.1 (28.9) | 23.8 (26.5) | | | 58.9 (25.8) | | | | 34.9 (19.1) | | | | 36.0 (29.5) | | | 36.9 (21.1) | | | 25.2 (30.8) | | | | 25.9 (31.5) | | | | 56.7 (24.0) | | | | 41.6 (26.0) |
| *p value* | *p=0.111* | *p=0.739* | *p=0.608* | | *p=0.384* | *p=0.948* | | | ***p=0.020*** | | | | *p=0.461* | | | | *p=0.064* | | | *p=0.078* | | | *p=0.671* | | | | *p=0.745* | | | | *p=0.449* | | | | *p=0.450* |
| ***Ethnic group*** |  |  |  | |  |  | | |  | | | |  | | | |  | | |  | | |  | | | |  | | | |  | | | |  |
| Kalenjin | 36.4 (15.9) | 30.5 (15.1) | 38.1 (13.5) | | 47.7 (20.9) | 17.5 (22.1) | | | 57.5 (20.6) | | | | 40.3 (12.5) | | | | 22.9 (18.2) | | | 38.1 (16.1) | | | 31.6 (32.7) | | | | 26.5 (27.5) | | | | 57.5 (16.5) | | | | 43.1 (19.2) |
| Kikuyu | 37.0 (16.7) | 28.5 (18.3) | 35.7 (15.2) | | 50.4 (19.3) | 17.7 (25.2) | | | 61.7 (17.4) | | | | 38.5 (11.7) | | | | 35.4 (19.8) | | | 43.5 (20.4) | | | 27.5 (31.0) | | | | 26.7 (28.6) | | | | 53.6 (19.5) | | | | 46.1 (19.0) |
| Luhya | 34.0 (19.1) | 24.8 (17.4) | 33.3 (21.5) | | 48.1 (24.4) | 24.8 (23.4) | | | 60.0 (22.4) | | | | 40.9 (19.0) | | | | 30.6 (25.9) | | | 37.5 (19.4) | | | 24.7 (29.6) | | | | 27.5 (29.4) | | | | 59.0 (24.0) | | | | 44.4 (27.3) |
| Teso | 27.6 (31.8) | 14.4 (21.9) | 24.0 (21.8) | | 48.1 (38.3) | 31.5 (34.1) | | | 76.6 (24.8) | | | | 21.3 (20.0) | | | | 43.8 (37.8) | | | 45.3 (29.0) | | | 29.3 (36.3) | | | | 29.3 (36.3) | | | | 56.5 (37.4) | | | | 38.6 (30.3) |
| Other | 29.6 (21.3) | 25.0 (15.8) | 25.8 (15.7) | | 55.1 (25.3) | 18.8 (24.4) | | | 62.9 (22.7) | | | | 28.9 (15.2) | | | | 26.2 (23.6) | | | 40.0 (15.6) | | | 21.3 (33.0) | | | | 17.6 (27.6) | | | | 64.4 (34.4) | | | | 42.4 (21.2) |
| *p value* | *p=0.172* | ***p<0.001*** | ***p=0.003*** | | *p=0.770* | ***p=0.048*** | | | ***p<0.001*** | | | | ***p<0.001*** | | | | ***p=0.004*** | | | *p=0.154* | | | *p=0.637* | | | | *p=0.571* | | | | *p=0.616* | | | | *p=0.681* |
| ***Marital status*** |  |  |  | |  |  | | |  | | | |  | | | |  | | |  | | |  | | | |  | | | |  | | | |  |
| Married | 33.4 (21.8) | 24.2 (18.6) | 32.4 (20.7) | | 47.9 (26.7) | 24.5 (26.0) | | | 65.1 (23.7) | | | | 36.2 (19.2) | | | | 32.5 (27.8) | | | 40.1 (20.8) | | | 24.5 (30.1) | | | | 25.9 (30.1) | | | | 57.9 (26.4) | | | | 42.0 (24.7) |
| Single | 32.3 (20.0) | 22.6 (19.0) | 30.7 (18.8) | | 53.5 (28.4) | 22.5 (27.1) | | | 65.8 (22.1) | | | | 35.5 (19.6) | | | | 32.6 (28.7) | | | 40.7 (21.8) | | | 33.9 (37.1) | | | | 30.1 (31.8) | | | | 58.2 (25.2) | | | | 47.5 (29.7) |
| separated/ widowed | 31.8 (19.5) | 26.6 (15.8) | 28.1 (18.2) | | 46.3 (19.5) | 21.3 (22.6) | | | 64.1 (17.0) | | | | 36.0 (18.2) | | | | 26.0 (18.5) | | | 32.8 (20.1) | | | 22.5 (28.0) | | | | 25.0 (28.1) | | | | 62.0 (18.6) | | | | 43.8 (27.0) |
| *p value* | *p=0.909* | *p=0.685* | *p=0.587* | | *p=0.336* | *p=0.781* | | | *p=0.544* | | | | *p=0.970* | | | | *p=0.594* | | | *p=0.302* | | | *p=0.112* | | | | *p=0.626* | | | | *p=0.806* | | | | *p=0.386* |
| ***Educational attainment*** | | | | | | |  | | | |  | | |  | | | |  | | | |  | | | |  | | | |  | | | |  | |
| No formal | 30.6 (20.0) | 23.1 (19.7) | 26.9 (18.5) | | 51.1 (26.3) | 20.3 (23.5) | | | 66.2 (19.3) | | | | 36.8 (17.6) | | | | 31.6 (25.2) | | | 38.8 (17.6) | | | 25.0 (35.8) | | | | 20.9 (28.8) | | | | 64.8 (28.2) | | | | 46.7 (26.4) |
| Primary | 34.0 (22.7) | 23.3 (18.1) | 33.6 (20.6) | | 50.9 (27.6) | 26.6 (27.3) | | | 54.7 (22.5) | | | | 36.0 (21.1) | | | | 33.5 (29.2) | | | 41.0 (21.7) | | | 25.9 (31.3) | | | | 26.6 (29.9) | | | | 60.1 (26.4) | | | | 43.0 (27.7) |
| Secondary + | 32.6 (19.4) | 25.7 (18.6) | 31.0 (19.9) | | 44.3 (24.7) | 20.8 (24.2) | | | 63.0 (23.0) | | | | 35.8 (16.0) | | | | 30.0 (25.4) | | | 37.9 (21.2) | | | 27.4 (30.4) | | | | 29.6 (31.4) | | | | 52.2 (21.9) | | | | 41.7 (22.1) |
| *p value* | *p=0.644* | *p=0.572* | *p=0.122* | | *p=0.135* | *p=0.138* | | | ***p<0.001*** | | | | *p=0.968* | | | | *p=0.611* | | | *p=0.496* | | | *p=0.899* | | | | *p=0.299* | | | | ***p=0.014*** | | | | *p=0.612* |
| ***Currently working*** | | | |  | | |  | | | |  | | |  | | | |  | | | |  | | | |  | | | |  | | | |  | |
| Yes | 33.4 (21.5) | 23.4 (18.1) | 31.9 (20.0) | | 49.2 (26.9) | 22.9 (25.5) | | | 62.6 (22.7) | | | | 35.8 (17.2) | | | | 30.8 (26.2) | | | 38.7 (20.5) | | | 25.5 (30.8) | | | | 26.5 (28.9) | | | | 59.0 (25.4) | | | | 42.0 (25.1) |
| No | 31.5 (20.3) | 27.2 (20.2) | 31.2 (21.5) | | 47.3 (25.5) | 29.3 (27.7) | | | 64.9 (24.4) | | | | 37.5 (26.9) | | | | 38.8 (32.7) | | | 45.1 (22.6) | | | 30.2 (35.7) | | | | 27.6 (36.9) | | | | 54.5 (27.0) | | | | 49.4 (29.4) |
| *p value* | *p=0.584* | *p=0.198* | *p=0.809* | | *p=0.662* | *p=0.115* | | | *p=0.533* | | | | *p=0.598* | | | | *p=0.066* | | | *p=0.052* | | | *p=0.347* | | | | *p=0.820* | | | | *p=0.295* | | | | *p=0.093* |
| ***Type of work*** |  |  |  | |  |  | | |  | | | |  | | | |  | | |  | | |  | | | |  | | | |  | | | |  |
| Agriculture | 35.5 (22.2) | 22.4 (15.9) | 31.7 (18.7) | | 49.6 (25,7) | 20.9 (24.1) | | | 65.3 (21.2) | | | | 35.8 (16.6) | | | | 32.3 (27.1) | | | 39.1 (19.1) | | | 24.1 (30.1) | | | | 25.2 (28.5) | | | | 59.3 (24.9) | | | | 41.6 (23.5) |
| Self-employed | 32.0 (20.8) | 24.7 (22.1) | 32.5 (22.8) | | 47.3 (29.5) | 23.9 (27.1) | | | 59.6 (24.0) | | | | 33.6 (18.9) | | | | 28.5 (25.7) | | | 38.3 (23.3) | | | 27.9 (32.2) | | | | 28.7 (29.4) | | | | 54.8 (27.6) | | | | 43.4 (28.8) |
| Unemployed | 30.0 (23.2) | 21.2 (17.7) | 29.4 (18.7) | | 51.7 (26.7) | 36.3 (32.0) | | | 67.7 (26.9) | | | | 38.2 (21.4) | | | | 31.5 (26.6) | | | 42.2 (24.0) | | | 23.3 (33.4) | | | | 26.7 (33.4) | | | | 71.5 (20.3) | | | | 42.7 (26.9) |
| Other | 27.6 (17.5) | 26.1 (18.1) | 31.9 (20.0) | | 50.4 (27.3) | 22.3 (23.7) | | | 53.5 (21.9) | | | | 39.0 (13.3) | | | | 28.7 (23.2) | | | 35.4 (19.0) | | | 27.8 (30.5) | | | | 27.8 (28.0) | | | | 59.1 (23.6) | | | | 40.4 (22.6) |
| *p value* | *p=0.525* | *p=0.672* | *p=0.928* | | *p=0.914* | *p=0.145* | | | ***p=0.035*** | | | | *p=0.587* | | | | *p=0.757* | | | *p=0.733* | | | *p=0.818* | | | | *p=0.914* | | | | *p=0.134* | | | | *p=0.953* |
| ***Monthly household income in past year*** | | | | | | | | | |  | | | | |  | | | |  | | | | |  | | | |  | | | |  | | | |
| <3,000 KSh | 34.9 (22.3) | 23.0 (17.8) | 31.8 (19.2) | | 51.6 (25.6) | 25.0 (26.9) | | | 67.9 (20.8) | | | | 37.7 (18.8) | | | | 34.8 (27.4) | | | 41.8 (20.9) | | | 25.2 (33.2) | | | | 26.8 (31.2) | | | | 61.3 (27.1) | | | | 43.2 (25.6) |
| 3,000 + KSh | 31.0 (20.5) | 24.8 (19.2) | 31.5 (21.8) | | 45.8 (27.9) | 23.0 (24.4) | | | 57.3 (24.6) | | | | 34.7 (19.8) | | | | 29.3 (27.7) | | | 37.5 (21.0) | | | 27.8 (30.4) | | | | 26.5 (29.8) | | | | 55.0 (23.3) | | | | 42.9 (26.5) |
| *p value* | *p=0.120* | *p=0.430* | *p=0.927* | | *p=0.067* | *p=0.523* | | | ***p<0.001*** | | | | *p=0.228* | | | | *p=0.090* | | | *p=0.082* | | | *p=0.493* | | | | *p=0.923* | | | | ***p=0.043*** | | | | *p=0.932* |
| ***Number of chronic conditions*** | | | | | | | | | | | | | | |  | | | |  | | | | |  | | | |  | | | |  | | | |
| 1 chronic condition | 34.1 (20.5) | 25.2 (18.0) | 32.4 (19.8) | | 50.4 (26.2) | 23.4 (25.4) | | | 63.0 (21.3) | | | | 35.0 (18.8) | | | | 28.6 (25.6) | | | 39.1 (19.9) | | | 23.9 (29.4) | | | | 24.4 (28.0) | | | | 55.6 (24.7) | | | | 42.5 (24.5) |
| 2+ chronic condition | 31.4 (22.5) | 22.1 (19.2) | 30.8 (20.8) | | 46.5 (27.2) | 24.6 (27.0) | | | 63.0 (25.5) | | | | 37.6 (19.6) | | | | 37.8 (29.4) | | | 40.7 (22.6) | | | 30.0 (34.6) | | | | 30.2 (33.4) | | | | 62.4 (26.6) | | | | 44.1 (27.9) |
| *p value* | *p=0.278* | *p=0.167* | *p=0.501* | | *p=0.221* | *p=0.694* | | | *p=0.987* | | | | *p=0.288* | | | | ***p=0.005*** | | | *p=0.525* | | | *p=0.106* | | | | *p=0.110* | | | | ***p=0.030*** | | | | *p=0.637* |
| ***Diabetes*** |  |  |  | |  |  | | |  | | | |  | | | |  | | |  | | |  | | | |  | | | |  | | | |  |
| Yes | 35.1 (23.3) | 26.0 (19.4) | 35.7 (21.4) | | 51.3 (27.2) | 26.5 (27.5) | | | 65.5 (24.1) | | | | 38.4 (20.3) | | | | 38.2 (29.5) | | | 41.4 (22.7) | | | 30.2 (35.0) | | | | 30.6 (33.1) | | | | 63.0 (26.4) | | | | 43.5 (26.7) |
| No | 31.8 (19.7) | 22.9 (17.8) | 29.2 (18.6) | | 47.4 (26.3) | 22.0 (24.7) | | | 61.7 (21.6) | | | | 34.5 (18.0) | | | | 27.7 (25.0) | | | 38.5 (19.6) | | | 23.7 (29.0) | | | | 23.5 (27.5) | | | | 54.6 (24.6) | | | | 43.2 (25.0) |
| *p value* | *p=0.189* | *p=0.153* | ***p=0.006*** | | *p=0.211* | *p=0.146* | | | *p=0.152* | | | | *p=0.108* | | | | ***p=0.001*** | | | *p=0.250* | | | *p=0.084* | | | | ***p=0.048*** | | | | ***p=0.007*** | | | | *p=0.931* |
| ***Number of drugs*** | | | | | | | |  | | | |  | | | |  | | | | |  | | | |  | | | |  | | | |  | | |
| 1 | 32.4 (20.4) | 23.3 (17.8) | 31.3 (20.3) | | 49.6 (26.2) | 22.9 (25.4) | | | 61.9 (22.5) | | | | 35.0 (19.2) | | | | 29.2 (26.2) | | | 39.1 (20.2) | | | 23.7 (29.8) | | | | 24.1 (28.6) | | | | 56.2 (24.5) | | | | 41.5 (25.0) |
| 2+ | 34.6 (23.8) | 26.2 (20.5) | 33.2 (19.8) | | 46.4 (27.8) | 27.0 (27.6) | | | 66.5 (24.2) | | | | 39.2 (18.6) | | | | 41.0 (29.6) | | | 42.0 (23.2) | | | 34.4 (35.7) | | | | 34.7 (34.0) | | | | 63.9 (28.1) | | | | 47.9 (27.8) |
| *p value* | *p=0.439* | *p=0.239* | *p=0.471* | | *p=0.376* | *p=0.243* | | | *p=0.135* | | | | *p=0.122* | | | | ***p=0.001*** | | | *p=0.310* | | | ***p=0.012*** | | | | ***p=0.009*** | | | | ***p=0.028*** | | | | *p=0.076* |
| ***Location of treatment when unwell*** | | | | | | | | | |  | | | | |  | | | |  | | | | |  | | | |  | | | |  | | | |
| County hospital | 31.8 (14.1) | 23.4 (15.0) | 28.9 (20.0) | | 42.6 (24.4) | 24.2 (26.7) | | | 58.4 (22.7) | | | | 40.0 (15.6) | | | | 29.9 (25.1) | | | 43.1 (22.8) | | | 23.4 (32.3) | | | | 35.2 (32.9) | | | | 63.0 (25.2) | | | | 47.0 (24.0) |
| Sub-county hospital | 34.3 (19.5) | 28.5 (17.9) | 34.6 (20.1) | | 52.0 (25.4) | 28.4 (24.9) | | | 64.5 (20.1) | | | | 39.3 (19.6) | | | | 33.6 (26.5) | | | 41.3 (18.4) | | | 27.2 (30.2) | | | | 32.1 (30.6) | | | | 62.1 (22.5) | | | | 45.5 (23.3) |
| Health centre | 31.2 (20.3) | 23.3 (18.4) | 32.2 (22.3) | | 48.1 (24.7) | 20.2 (23.8) | | | 62.6 (24.0) | | | | 33.5 (19.3) | | | | 33.0 (27.5) | | | 36.9 (20.5) | | | 26.9 (32.4) | | | | 23.1 (29.7) | | | | 56.9 (26.8) | | | | 39.9 (26.9) |
| Dispensary | 32.9 (26.9) | 18.6 (20.2) | 27.3 (15.9) | | 49.0 (32.5) | 22.9 (29.1) | | | 64.7 (25.5) | | | | 30.8 (17.6) | | | | 30.2 (30.8) | | | 40.9 (22.3) | | | 22.3 (30.7) | | | | 19.7 (25.6) | | | | 53.3 (29.1) | | | | 42.2 (29.6) |
| Private provider | 40.4 (23.1) | 26.5 (16.6) | 38.1 (22.8) | | 47.3 (22.0) | 21.9 (28.7) | | | 57.5 (22.9) | | | | 44.4 (23.1) | | | | 30.4 (23.2) | | | 34.3 (28.6) | | | 41.1 (37.5) | | | | 28.6 (30.8) | | | | 53.0 (19.3) | | | | 43.5 (22.0) |
| *p value* | *p=0.590* | ***p=0.021*** | *p=0121* | | *p=0.526* | *p=0.303* | | | *p=0.579* | | | | ***p=0.021*** | | | | *p=0.920* | | | *p=0.375* | | | *p=0.349* | | | | ***p=0.034*** | | | | *p=0.203* | | | | *p=0.617* |
| ***Has NHIF cover*** | | | | | |  | | |  | | | |  | | | |  | | |  | | |  | | | |  | | | |  | | | |  |
| Yes | 32.1 (21.3) | 24.7 (18.6) | 31.1 (20.9) | | 47.4 (25.5) | 21.5 (25.6) | | | 53.1 (25.7) | | | | 39.6 (21.0) | | | | 29.1 (26.6) | | | 37.8 (20.8) | | | 26.7 (33.7) | | | | 27.4 (32.9) | | | | 55.9 (23.6) | | | | 39.7 (24.8) |
| No | 33.3 (21.3) | 23.8 (18.6) | 32.0 (20.0) | | 49.3 (27.0) | 24.5 (26.0) | | | 66.3 (21.1) | | | | 44.3 (26.2) | | | | 33.0 (27.8) | | | 40.4 (21.1) | | | 26.1 (31.0) | | | | 26.4 (29.4) | | | | 59.1 (26.3) | | | | 44.3 (26.2) |
| *p value* | *p=0.665* | *p=0.715* | *p=0.764* | | *p=0.604* | *p=0.376* | | | ***p<0.001*** | | | | *p=0.104* | | | | *p=0.287* | | | *p=0.355* | | | *p=0.897* | | | | *p=0.806* | | | | *p=0.372* | | | | *p=0.209* |

Note. p-values in bold: statistically significant difference between response items
