# Supplementary material for: Understanding the treatment burden of people with chronic conditions in Kenya: A cross-sectional analysis using the Patient Experience with Treatment and Self-Management (PETS) questionnaire
Source: PLOS Glob Public Health. 2023 Jan 17;3(1):e0001407. doi: 10.1371/journal.pgph.0001407 (PMC10021888; doi:10.1371/journal.pgph.0001407)
Supplement: S7 Table — (DOCX) [file pgph.0001407.s008.docx]

##

## **S7 Table.** **PETS scores by number of chronic conditions, total sample**

| **PETS scale** | **1 chronic condition** (standard error mean) | **2+ chronic conditions** (standard error mean) | **p-value*** |
| --- | --- | --- | --- |
| Medical information | 34.1 (1.50) | 31.4 (2.09) | 0.278 |
| Medications | 25.2 (1.34) | 22.1 (1.79) | 0.167 |
| Medical appointments | 32.4 (1.46) | 30.8 (1.93) | 0.501 |
| Monitoring health | 50.4 (1.95) | 46.5 (2.53) | 0.221 |
| Interpersonal challenges | 23.4 (1.86) | 24.6 (2.50) | 0.694 |
| Medical and health care expenses | 63.0 (1.56) | 63.0 (2.37) | 0.987 |
| Difficulties with health care services | 35.0 (1.52) | 37.6 (1.90) | 0.288 |
| Role/social activity limitations | 28.6 (1.88) | 37.8 (2.73) | **0.005** |
| Physical/mental fatigue | 39.1 (1.46) | 40.7 (2.10) | 0.525 |
| Bother due to reliance on medicine | 23.9 (2.19) | 30.0 (3.23) | 0.106 |
| Bother due to side effects of medicine | 24.4 (2.08) | 30.2 (3.11) | 0.110 |
| Diet | 55.6 (1.91) | 62.4 (2.52) | **0.030** |
| Exercise and physical therapy | 42.5 (1.92) | 44.1 (2.68) | 0.637 |

Note. * Independent samples t-test, 2-sided
