# Supplementary material for: Understanding the treatment burden of people with chronic conditions in Kenya: A cross-sectional analysis using the Patient Experience with Treatment and Self-Management (PETS) questionnaire
Source: PLOS Glob Public Health. 2023 Jan 17;3(1):e0001407. doi: 10.1371/journal.pgph.0001407 (PMC10021888; doi:10.1371/journal.pgph.0001407)
Supplement: Attachment — Submitted filename: PGPH-D-22-01437 Authors response 28Nov2022.docx [file pgph.0001407.s009.docx]

| **Comment** | **Author response** *(‘revised manuscript’ refers to manuscript with changes tracked)* |
| --- | --- |
| ***Reviewer 1*** | |
| My major concern with the manuscript is regarding the translation and the cultural adaptation of the Patient Experience with Treatment and Self-Management (PETS) questionnaire.  A section in the manuscript titled “Adaptation of the PETS instrument” briefly mentions the steps undertaken by the team to translate the questions into Swahili.  But I feel it would be more appropriate to publish a full translational study of the Kenyan version initially elaborating on the process of translation and validation before this study. | Thank you for this comment. We agree with the Reviewer that a separate validation of the translated version of the PETS would have been desirable. But doing so would have required time, and we would not have had a translated version of a treatment burden measure available for the PIC4C study. To not include such a key outcome would have been suboptimal. We therefore decided to pursue translation of an existing measure and cognitively test it with a small patient sample to verify its face validity with the target sample.  We went through a very rigorous process of translating and adapting the PETS before implementing the tool, which we describe in the paper. But we agree with the Reviewer that the paper would benefit from a more detailed description of the translation and we have now amended the text in the main manuscript (p. 6-7, lines 134-163 in the revised manuscript) and have added a new Figure to the supplementary materials (S1 Figure) describing the individual steps taken. |
| ***Reviewer 2*** | |
| 1. Please do rationale of choosing two place where the study has been conducted. | Thank you for raising this point. The study setting (Western Kenya: Busia and Trans Nzoia counties) was determined by the ‘Primary Health Integrated Care Project for Chronic Conditions’ (PIC4C), which provided the context for our study. PIC4C was set within the Academic Model Providing Access to Health Care (AMPATH) in western Kenya and launched in 2018 by the Kenyan Ministry of Health in partnership with AMPATH/Moi, Access Accelerated and the World Bank. We have cited a reference to the study protocol, which describes the PIC4C model in greater detail, in the Methods section but agree that this may not have been sufficiently clear. We have now added the following to the first paragraph of the Methods section (p. 5, lines 105-107) of the revised manuscript) to clarify this:  *“PIC4C was launched in 2018 by the Kenyan Ministry of Health in partnership with AMPATH/Moi, Access Accelerated and the World Bank. The model was piloted in Busia and Trans Nzoia counties in Western Kenya, and which formed the location for the present study.”* |
| 2. How the sample size has been estimated? Give the statistical tools used to estimate the sample | Thank you for highlighting this important point. We should clarify that we did not use a formal statistical approach to estimate the sample size. Our approach is more akin to a non-probability sample in the sense that our aim was not to test a hypothesis about a broad population (by means of using a statistical sample), but to develop an initial understanding of treatment burden in a population diagnosed with hypertension and/or diabetes in two counties in Western Kenya. We highlight this in the ‘Strengths and Limitations’ section of the Discussion (p. 22, lines 430-434 of the revised manuscript), where we note that *“Our sample was not representative of the patient population in Busia and Trans Nzoia counties and we cannot, therefore, generalise across the wider patient population in either county. However, this was not the aim of this survey. Indeed, the sample was meant to capture people using PIC4C services with targeted sampling of those with hypertension and/or diabetes specifically.”*  In the absence of any formal measure of treatment and self-management burden in our target population, we were guided by the sample size used by the developers of the PETS tool. We should further add that the PETS further served as a basis to identify a subsample of people with hypertension and/or diabetes for further in-depth interviews (not reported in this study). We have amended the introductory text in the Methods to clarify this point (p. 5-6, lines 108-117 of the revised manuscript):  *“One key component of our study was to assess understand the treatment and self-management burden among a group of people diagnosed with hypertension and/or diabetes since PIC4C implementation and how the burden is distributed across a range of socio-demographic characteristics. We used the Patient Experience with Treatment and Self-Management (PETS) instrument [21] as it assess treatment burden in patients with chronic health conditions requiring self-management. This comprehensive measure allows understanding what aspects of treatment and self-management prove to be most burdensome to people with chronic conditions in Kenya. The survey served as a basis from which to recruit a subsample of people with hypertension and/or diabetes for further explore treatment burden using in-depth interviews.”* |
| 3. Please add PETS questionnaire in the supplementary. | We are not permitted to include the PETS in the supplementary materials as the PETS, including all modified, translated, and adapted versions of it, are protected by copyright, ©2020 Mayo Foundation for Medical Education and Research. The PETS can be made available upon request and we added the following note to the Methods section (p. 7, lines 160-163 of the revised manuscript):  *“The English language PETS and its final translated version can be made available upon written request to Dr Eton, the principal developer of the measure, at dteton99@gmail.com.”* |
| 4. Figures and not so visible, revise | We are uncertain what precisely the Reviewer wishes us to change regarding the Figures We are happy amend figures as guided by the editorial team. |
| ***Reviewer 3*** | |
| The study evaluates an important perspective on patient centered research for NCDs. The authors have substantially highlighted the limitations of the study. Why this manuscript may be the first to utilise the approach stated in the methods, I am of the opinion that such a statement declaring "this is the first paper..." may be more subtly made. There could other similar research approaches that authors may not be aware of. Hence, if such declaration is deemed necessary, the word may could prefix the statement. | Thank you very much for your supportive comments on our manuscript. Following the Reviewer’s suggestion we have revised the sentence as follows (Discussion, first paragraph, p. 20, line 350):  *“To our knowledge, this is one of the first studies that have…”* |
| ***Reviewer 4*** | |
| This is an interesting article that moves away from the assessment of disease burden and mortality to treatments that can become a significant burden in the management of chronic diseases for patients' quality of life. | Thank you very much for your supportive comments on our manuscript. |
| It includes a validation component of PETS in the Kenyan context which was well described by the authors. But have the steps been documented? What was the level of concordance between translators at the different stages to better capture the challenge of the adaptation steps ? (I encourage the authors to publish this data in particular to help other teams to better prepare for this stage) | Thank you for raising this. We have now amended the text in the Methods section to clarify the process (p. 6-7, lines 134-163 in the revised manuscript); we have also added a new Figure to the supplementary materials (S1 Figure) describing the individual steps taken.  It is not possible to make the documents produced during the translation process publicly available (e.g., as additional supplementary materials). The main reason is that the PETS, including all modified, translated, and adapted versions of it, are protected by copyright, ©2020 Mayo Foundation for Medical Education and Research. The PETS can be made available upon request and we added the following note to the Methods section (p. 7, lines 160-163 of the revised manuscript):  *“The English language PETS and its final translated version can be made available upon written request to Dr Eton, the principal developer of the measure, at dteton99@gmail.com.”* |
| 1. Can the participation rate be clarified ?  Could you say - we identified ## eligible participants and approached them in a random order with the aim of recruiting n=150 in each county. When we reached this number we ceased to approach further potential participants. | We oversampled to enable random selection of n=150 participants in each county (Busia: 202; Tranz Nzoia: 213). We stopped recruiting once the targeted/actual sample of n=150 per county was achieved. We amended the text as follows (Methods; p. 8-9, lines 187-194 of the revised manuscript; addition underlined):  *“Anticipating non-response of 30-45%, we oversampled to around 200 in each county (Busia: 202; Tranz Nzoia: 213) so as to randomly select 150 to be surveyed in each. The study population included all patients who had been screened and had hypertension and/or diabetes confirmed through PIC4C service efforts between 2018 and 2020. We used the permanent and the daily registers held at individual health facilities to identify eligible patients. If the randomly selected patient was not available or did not consent, we moved to the next patient on the list of randomly selected participants. This process was repeated until the required sample in each cluster was achieved.”* |
| 2. 60 minutes for a survey, especially by phone is not easy. How could fatigue have affected the quality of responses? | Thank you for drawing our attention to this issue. To clarify: all interviews were conducted face-to-face. We only used the phone as a means to contact and invite potential participants. We have now clarified this in the main text, noting that *“For practical reasons, the survey was conducted in-person at a health facility closest to the invited participants’ homes.”* (p. 9, lines 201-202 of the revised manuscript)  We should highlight that the average duration of 60 minutes includes breaks where survey participants requested this so as to minimise the risk of participants getting fatigued. We have clarified this in the text (p. 9, lines 208-209 of the revised manuscript). |
| 3. "The majority of respondents reported having at least one chronic condition" : as they were selected on the basis of having at least one chronic disease, 100% is expected. So can we delete that sentence or rephrase it? | We agree with the Reviewer and have rephrased the sentence as follows (p. 11, line 248 of the revised manuscript):  *“The majority of respondents reported having one chronic condition;…”* |
| 4. The paragraph "Implications for practice and research" can be further clarified to clearly define the contribution of this work to change. Specify areas where investment needs to be strengthened for each of the communities and perhaps identify other factors that would be vulnerable to actions. | Thank you for your comment. We have revised the Discussion section to further clarify study implications (p. 23-24, lines 457-488 of the revised manuscript). |
| 5. The conclusion (abstract and manuscript) should be more specific to describe the burden of treatment, the associated factors identified and open up on perceived actions as a response to improve the situation | Thank you for your comment. We agree that the conclusions could be more specific regarding its implications and have amended the abstract as follows (p. 3, lines 57-60 of the revised manuscript):  *“**This study provides important insights for Kenya and similar settings where governments are working to achieve universal health coverage. It highlights the importance of financial protection not only to prevent the economic burden of seeking healthcare for chronic conditions but also to reduce the associated treatment burden.”* |
| ***Reviewer 5*** | |
| 1. The authors state that the objective of the paper was to explore treatment burden among people with NCDs in Kenya. Being stated this, the authors go on at length to explain the validation of the instrument (PETS), they have adapted to the Kenyan context. I am quite confused whether the authors want to present the validation of the instrument or the actual application of the survey instrument to assess the burden. Please explicitly state this in your objectives. | Thank you for raising this point. You are correct that the primary aim of the paper was to explore treatment burden among people with NCDs in Kenya. However, given that we used an instrument that was developed in the USA that, at least to our knowledge, has not yet been used in the Kenyan context, we felt it important to describe the process of adapting the PETS. We are happy to shorten the description of the process in the text but note that Reviewer 1 and Reviewer 4 both asked for further detail. We have thus included additional material in the Supplement and in doing so we hope to have addressed related concerns adequately. |
| 2. What was the rationale for choosing the particular instrument (PETS) to capture treatment burden? | Thank you for raising this important point. There are a number of instruments that measure (aspects of) treatment burden, such as the Treatment Burden Questionnaire (TBQ) or the Multimorbidity Treatment Burden Questionnaire (MTBQ), which we cited in the Discussion. We opted for the PETS since it is the only validated instrument that measures both treatment *and* self-management burden. The successful management of chronic conditions such as diabetes or hypertension requires active management by patients of their condition. However, as we highlight in the introduction to the paper, self-management tasks can pose a significant additional burden to patients that can, ultimately undermine the effective management of chronic conditions.  We have now clarified the relevant text as follows (Methods; p. 5-6, lines 108-117 of the revised manuscript, addition underlined):  *“One key component of our study was to assess the prevalence of the treatment and self-management burden among a group of people diagnosed with hypertension and/or diabetes since PIC4C implementation, and to understand the distribution of the burden across a range of socio-demographic characteristics. We used the Patient Experience with Treatment and Self-Management (PETS) instrument [21] as it assess treatment burden in patients with chronic health conditions requiring self-management.* *This comprehensive measure allows understanding what aspects of treatment and self-management prove to be most burdensome to people with NCDs in Kenya*.” |
| 3. In the participant recruitment section, I am not quite clear with the sample size calculations. The authors have used a SS of 300 and the rationale for this is stated as “this ss was used by the developers of the PETS survey instrument to test its validity”, but that cannot be the rationale here since you are using this sample to assess the treatment burden. | Thank you for highlighting this important point. We should clarify that we did not use a formal statistical approach to estimate the sample size. Our approach is more akin to a non-probability sample in the sense that our aim was not to test a hypothesis about a broad population (by means of using a statistical sample), but to develop an initial understanding of treatment burden in a population diagnosed with hypertension and/or diabetes in two counties in Western Kenya. We highlight this in the ‘Strengths and Limitations’ section of the Discussion (p. 22, lines 430-434 of the revised manuscript), where we note that *“Our sample was not representative of the patient population in Busia and Trans Nzoia counties and we cannot, therefore, generalise across the wider patient population in either county. However, this was not the aim of this survey. Indeed, the sample was meant to capture people using PIC4C services with targeted sampling of those with hypertension and/or diabetes specifically.”*  In the absence of any formal measure of treatment and self-management burden in our target population, we were guided by the sample size used by the developers of the PETS tool. We agree with the Reviewer that the developers used this sample size for validating the instrument rather than assessing treatment burden as such. We should further add that the PETS further served as a basis to identify a subsample of people with hypertension and/or diabetes for further in-depth interviews (not reported in this study). We have added the following sentence to the introductory text in the Methods to clarify this point (p. 5-6, lines 115-117 of the revised manuscript):  *“The survey served as a basis from which to recruit a subsample of people with hypertension and/or diabetes for further explore treatment burden using in-depth interviews.”* |
| 4. Why did the authors oversample? Did you expect non-response and how much was this percentage ? | Oversampling was informed by previous studies within AMPATH and the experience of PIC4C implementation. Based on this, we expected non-response rate of between 30-45% using registers and telephone for recruitment. We therefore oversampled at 34.5% for Busia and 42% for trans Nzoia. We have amended the corresponding text as follows (Methods; p. 8-9, lines 187-194 of the revised manuscript; addition underlined) (see also our response to comment 6 below):  *“Anticipating non-response of 30-45%, we oversampled to around 200 in each county (Busia: 202; Tranz Nzoia: 213) so as to randomly select 150 to be surveyed in each. The study population included all patients who had been screened and had hypertension and/or diabetes confirmed through PIC4C service efforts between 2018 and 2020. We used the permanent and the daily registers held at individual health facilities to identify eligible patients. If the randomly selected patient was not available or did not consent, we moved to the next patient on the list of randomly selected participants. This process was repeated until the required sample in each cluster was achieved.”* |
| 5. How did the authors randomly select the patients from the study population? | We have amended the corresponding text as follows (Methods; p. 8, lines 176-179 of the revised manuscript; addition underlined):  *“Our sampling strategy was based on random sampling of PIC4C facilities in the two counties. In addition, random sampling was done at each strata, with stratification based on the location (rural/urban) or level (size) and then at the facility-level based on chronic condition, gender and age.”* |
| 6. The authors have stated they used the register as the sampling frame but patients from what period registered in the facilities were selected. And what if the randomly selected patient was not available or did not consent to the study. | Thank you for this comment. The period was not more than 2 years since confirmed diagnosis of the health condition. We therefore selected patients with year of diagnosis 2018-2020 and current clinical attendance. If the randomly selected patient was not available or did not consent, we moved to the next patient on the list of randomly selected participants. This process was repeated until the required sample in each cluster was achieved. We have amended the text as follows (Methods; p. 8-9, lines 189-194 of the revised manuscript; addition underlined):  *“The study population included all patients who had been screened and had hypertension and/or diabetes confirmed through PIC4C service efforts between 2018 and 2020. We used the permanent and the daily registers held at individual health facilities to identify eligible patients. If the randomly selected patient was not available or did not consent, we moved to the next patient on the list of randomly selected participants. This process was repeated until the required sample in each cluster was achieved.”* |
| 7. In the analysis section, its not clearly stated the individual scoring of each item, and the minimum and maximum scoring. Why was the scale score transferred to 0 to 100 scale and what does 0 means and what does 100 means? | Response scales vary across PETS domains (i.e., there is no single response scale used for all domains). Furthermore, the number of items is not the same across all of the PETS domain scales. Therefore, to facilitate interpretability, all raw scale scores are converted to a standard 0 to 100 metric with 0 indicating ‘no burden’ and 100 indicating ‘highest burden.’ Higher scores on any PETS domain scale always indicate higher burden. We added the following sentence (Methods; p. 10, lines 221-226 of the revised manuscript):  *“As response scales vary across PETS domains (i.e., there is no single response scale used for all domains) and the number of items is not the same across all of the PETS domain scales, we converted all raw scale scores to a standard 0 to 100 metric to facilitate interpretability, with 0 indicating ‘no burden’ and 100 indicating ‘highest burden.’ Higher scores on any PETS domain scale always indicate higher burden.”* |
| 8. Can the results section first describe the validation if the authors want to include that also as the objective of the paper and then in the next section describe the finding of the survey using the validated instrument. | Please see our response to point 1 above. |
| 9. In the results section, suddenly the authors mention about 2 single item scale, why was this included? Was the validity of it checked? Can this be mentioned in the methodology. | Thank you for drawing our attention to this oversight. The PETS vs. 2.0 contains 12 multi-item and two single-item scales. The single-item scales assess respectively bother due to medication side effects and bother due to having to rely on medication. They are found in the previously validated English-language version of the PETS. We have amended the text in the Methods section accordingly (Methods; p. 6, lines 125-131 of the revised manuscript, changes underlined):  *“It contains a series of 12 multi-item and two single item scales: (1) medical information, (2) medications, (3) medical appointments, (4) monitoring health, (5) diet, (6) exercise or physical therapy, (7) medical equipment, (8) relationships with others, (9) medical and health care expenses, (10) difficulty with health care services, (11) role and social activity limitations, and (12) physical and mental exhaustion. The single-item scales assess respectively bother due to medication side effects and bother due to having to rely on medication.”*  We note an error in Table 2 of our manuscript: the two medication bother items are separate scales and should have not been included in Table 2. We have now rectified this error (see Results Table 2, p. 14 of the revised manuscript).  Your comment has also alerted us to one other point that we believe was not made sufficiently clear in the original manuscript. We did not report on the domain ‘medical equipment’ since only 5% of survey respondents (n=14) reported using medical equipment that would enable self-monitoring at home. We have now added the following to the Methods section to clarify this point (p. 9, lines 209-211 of the revised manuscript):  *“The only exception was the domain ‘medical equipment’, which was only completed by 5% (n=14) survey respondents. We therefore did not consider this domain in the further analysis.”* |
